# Climate‐driven changes in macrobenthic communities in the Mediterranean Sea: A 10‐year study in the Bay of Banyuls‐sur‐Mer

**DOI:** 10.1002/ece3.5569

**Published:** 2019-08-29

**Authors:** Paulo Bonifácio, Antoine Grémare, Jean-Michel Amouroux, Céline Labrune

**Affiliations:** ^1^ CNRS, Environnements et Paléoenvironnements Océaniques et Continentaux (EPOC), UMR 5805 Université de Bordeaux Talence France; ^2^ CNRS, Laboratoire d'Ecogéochimie des Environnements Benthiques (LECOB), UMR 8222, Observatoire Océanologique Sorbonne Université Banyuls-sur-Mer France

**Keywords:** climate change, Gulf of Lions, North Atlantic Oscillation, Temporal changes, Western Mediterranean Oscillation, Zoobenthos

## Abstract

Marine ecosystems worldwide are affected by both natural variation and human activities; to disentangle and understand their individual role in influencing the macrobenthic community composition is challenging. The relationship between interannual variability in atmospheric circulation, dictated by the climatic oscillation indices, and the benthic macrofauna composition was assessed at four sampling sites located in the Bay of Banyuls‐sur‐Mer (NW Mediterranean Sea). Between 2004 and 2013, these sites were sampled annually during autumn/winter and analyzed for sediment grain‐size and benthic macrofauna composition (species richness, abundance, and biomass). Temporal changes in these descriptors were correlated with two climatic indices (NAO and WeMO indices) and a set of environmental parameters integrated over three different time periods (i.e., whole year, springtime, and wintertime). Our results confirm the occurrence of major temporal changes in the composition of macrobenthic communities within the Gulf of Lions. More specifically, the results indicate that (a) the WeMO appears to be more closely related to benthic macrofauna composition in the Bay of Banyuls‐sur‐Mer than the NAO, (b) winter is a better integration period than spring or the whole year as a proxy for community composition changes, and (c) Rhône River water flow is likely involved in the control of benthic macrofauna composition in the whole Gulf of Lions. The present study highlights the importance of WeMO as a regional proxy, which can be used to evaluate changes in benthic macrofauna linked to climatic variability.

## Graphical abstract

This study is the first which establishes a relationship between a recent and regional climatic oscillation (WeMO) and benthic macrofauna communities. The results show responses to climate changes in all ecological levels (individual species to community level) based on 10‐year series.

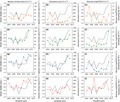

## 1 INTRODUCTION

As the impact of human activities on marine ecosystems is increasing, ecological assessment is becoming a central topic for the management of European seas (De Backer, Van Hoey, Coates, Vanaverbeke, & Hostens, 2014; MSFD, 2008; Vačkář, ten Brink, Loh, Baillie, & Reyers, 2012; WFD, 2000). In order to assess the extent to which human activities impact natural systems, it is essential to distinguish anthropogenic impacts from natural (e.g., climatic) variability (Hewitt, Ellis, & Thrush, 2016). The North Atlantic Oscillation (NAO) is the major source of interannual variability in the atmospheric circulation in the North Atlantic (Hurell, 1995). The NAO largely controls local changes in a large set of meteorological parameters such as water temperature, salinity, wind strength/direction, and storms. The NAO index provides a good summary of general weather patterns influencing marine ecosystems and affecting the abundance, biomass, growth, and survival rates of marine organisms (Drinkwater et al., 2003; Fromentin & Planque, 1996; Kröncke, Dippner, Heyen, & Zeiss, 1998; Shojaei et al., 2016). Several studies have highlighted the consequences of changes in meteorological parameters and, thus, of NAO on (a) zooplankton communities in the western Mediterranean (Fernández de Puelles, Valencia, & Vincent, 2004); the North Atlantic and the North Sea (Fromentin & Planque, 1996); (b) fisheries in the NW Mediterranean Sea (Lloret, Lleonart, Sole, & Fromentin, 2001); (c) recruitment of anchovy (Santojanni et al., 2006) in the Adriatic Sea; (d) physical condition of migratory bullet tuna stock during pre‐ and postreproductive movement (Muñoz‐Expósito et al., 2017) in the western Mediterranean; and (e) benthic macrofauna composition in the North Sea (Hagberg & Tunberg, 2000; Kröncke et al., 1998, 2011; Kröncke, Zeiss, & Rensing, 2001; Rees et al., 2006; Shojaei et al., 2016; Tunberg & Nelson, 1998). Between 1978 and 1995, Kröncke et al. (1998) seasonally sampled five sites located between 12 and 20 m depth off the Island of Norderney. The study demonstrated that the abundance and species richness of benthic macrofauna sampled between April and July were correlated significantly with the NAO index. The authors suggested that the mediator between NAO and benthic macrofauna was sea surface temperature (SST) in late winter and early spring. This SST‐driven hypothesis was supported by further observations by Beukema (1985) who reported the decrease in *Echinocardium cordatum* populations after severe winters.

Lloret et al. (2001) were the first to correlate climatic oscillations with biological parameters in the NW Mediterranean and studied the relationship between fish and invertebrate landings, the Rhône and Ebre River water flow, and the NAO index. Findings from the study included (a) a negative correlation between water flow of these two rivers and the NAO, and (b) a positive correlation between the landings of 13 species of fishes and invertebrates and water flow. The authors suggested a link between recruitment and local environmental conditions such as river discharge, wind speed and direction, and global environmental conditions (i.e., NAO). However, recent studies in the NW Mediterranean have focused on the Western Mediterranean Oscillation index (WeMO index) rather than the commonly used NAO index as a proxy of local climatic variability (Martín, Sabatés, Lloret, & Martin‐Vide, 2012; Martin‐Vide & Lopez‐Bustins, 2006; Martin‐Vide et al., 2008). These two indices do not correlate significantly when standardized on an annual basis or in wintertime (Martín et al., 2012; Martin‐Vide & Lopez‐Bustins, 2006). The WeMO index has been shown to be more relevant than the NAO index to account for monthly precipitation anomalies in the Iberian Peninsula (Martin‐Vide & Lopez‐Bustins, 2006; Martin‐Vide et al., 2008). Further, Martín et al. (2012) showed that positive WeMO index values correlated significantly with low SST and high river run‐offs, which have a significant positive effect on sardine and anchovy landings per unit effort. Conversely, and based on a 45‐year time series, Keller, Valls, Hidalgo, and Quetglas, (2014) did not show any influence on the landings of *Sepia officinalis* in the western Mediterranean by either the NAO or the WeMO index, but only by SST.

Most of the benthic macrofauna data available in the Gulf of Lions (Bonifácio et al., 2018; Grémare, Amouroux, & Vétion, 1998; Grémare, Sardá, et al., 1998; Labrune, Grémare, Amouroux, et al., 2007; Labrune, Grémare, Amouroux, et al., 2007; Labrune, Grémare, Guizien, & Amouroux, 2007; Labrune et al., 2008; Massé, 2000; Salen‐Picard, 1981) have been collected over too narrow time scales to soundly assess their correlation with climatic oscillations whereas few studies by achieving long‐term comparisons. In 1967/68, Guille (1970) first described the benthic macrofauna communities of soft‐bottom habitats of the Catalan French coast. Grémare, Amouroux, and Vétion (1998) then demonstrated the occurrence of major changes in both sediment grain‐size and macrofauna composition between 1967/68 and 1994, and suggested that these changes were due to the decrease in fine particles most likely caused by an increase in the frequency of easterly storms. By using a different procedure to assess resuspension events, Labrune, Grémare, Guizien, et al. (2007) suggested that positive NAO index periods were related to low frequency of strong resuspension events and high abundance and biomass of benthic fauna. The underlying hypothesis put forward from this work was that the low frequency of resuspension events, especially during springtime, contributes to favorable conditions, thus resulting in a good recruitment of benthic macrofauna. Furthermore, they suggested that the positive periods of NAO index would indirectly and positively affect the abundance of benthic macrofauna as observed for the polychaete *Ditrupa arietina*, one of the most abundant species found in sandy sampling sites during 1994 and 2003. Later, Bonifácio et al. (2018) compiled and compared *D. arietina* abundances along the Gulf of Lions recorded between 1989 and 2013 (*N* = 17) and NAO and WeMO indices. Results inferred that NAO may have more influence on the recruitment of *D. arietina* than WeMO in the Gulf; but, they highlighted the urgent need for a long‐term monitoring series in order to confirm their hypothesis. In this context, the main objective of this study was (a) to assess changes in sediment grain‐size and benthic macrofauna composition based on data collected annually from 2004 to 2013 in the Bay of Banyuls‐sur‐Mer and (b) to evaluate the relationship between these changes with NAO and WeMO indices, and the main environmental parameters which are affecting the NW Mediterranean Sea. To achieve these objectives, the present study focuses on benthic macrofauna composition at four sampling sites, which are representative of the main benthic communities described by Guille (1970).

## 2 MATERIALS AND METHODS

### 2.1 Study area and sampling sites

**Figure 1:**
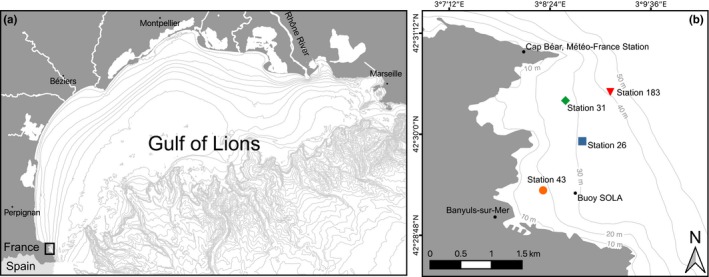
Delimitation of the study area in the (a) Gulf of Lions and (b) location of the four sampling sites within the Bay of Banyuls‐sur‐Mer. Symbols indicate sampling sites: 43 (circle), 31 (diamond), 26 (square), and 183 (triangle)

The Bay of Banyuls‐sur‐Mer is located within the Gulf of Lions in the northwestern Mediterranean Sea (Figure 1). Within this bay, four sites were sampled once a year between 2004 and 2013 (Table 1). Sites were chosen to represent the main benthic communities described by Guille in 1968 (Guille, 1970; Labrune, Grémare, Guizien, et al., 2007), whereby community names were maintained in accordance with those described by Guille (1970). Sites were sampled for sediment grain‐size analysis and benthic macrofauna during the end of autumn/beginning of winter (November–December) on board the RV *Nereis* II.

**Table 1:** Location (WGS84, degrees, and decimal minutes) and depth of the four sampling sites in each benthic community

Sampling site	Community	Latitude (N)	Longitude (E)	Depth (m)	Sediment
43	*Spisula subtruncata*	42°29.33′	03°08.32′	15	Well‐sorted fine sand
31	*Nephtys hombergii*	42°30.40′	03°08.59′	26	Muddy sand
26	*Scoloplos armiger*	42°29.92′	03°08.79′	31	Sandy mud
183	*Venus ovata*	42°30.50′	03°09.11′	43	Mud

Note

Sediment classification followed the original classification of Aloisi, Got, and Monaco (1973) which was revised by Labrune, Grémare, Guizien, et al. (2007).

### 2.2 Grain‐size analysis

At each sampling site, a 0.1 m^2^ van Veen grab was taken for sediment grain‐size analysis. Sediment grain‐size analysis was performed on fresh sediment using a Malvern Mastersizer^®^ 2000 laser microgranulometer and expressed as median grain diameter (*D*_0.5_) and in volume percentages of grain‐size fractions (<30, 30–63, 63–250, 250–500, 500–2,000 µm). Sediment grain‐size data are lacking for sampling site 183 during 2005 and 2006.

### 2.3 Benthic macrofauna

Five replicate grab samples were taken per sites for faunal analysis, immediately sieved on a 1 mm mesh, and fixed with 5% formalin buffered in seawater. At the laboratory, macrofauna was sorted, identified to the lowest possible taxonomic level (most often to species level), and counted. Biomass was assessed by measuring the weight loss after combustion (450°C, 5 hr) of dried samples.

### 2.4 Climatic indices

#### 2.4.1 North Atlantic Oscillation

The North Atlantic Oscillation is responsible for changes in the trajectories of surface westerlies across the North Atlantic toward Europe (Hurrell, 1995). Such changes can be described through several indices of NAO estimated using different approaches. During the present study, we used the classical NAO index developed by Hurrell and Deser (2009) based on the principal component (PC) time series of the leading empirical orthogonal function (EOF) of Sea Level Pressure anomalies over the Atlantic area (20°–80°N, 90°W–40°E). This method presents better representations of the full spatial patterns of the NAO. Positive values are typically associated with stronger‐than‐average westerlies and storms over northern Europe and milder weather with less‐than‐average storms over western Europe and the Mediterranean Sea. Corresponding data were provided by the Climate Analysis Section (NCAR, Boulder, USA, https://climatedataguide.ucar.edu/climate-data/hurrell-north-atlantic-oscillation-nao-index-pc-based).

#### 2.4.2 Western Mediterranean Oscillation

**Figure 2:**
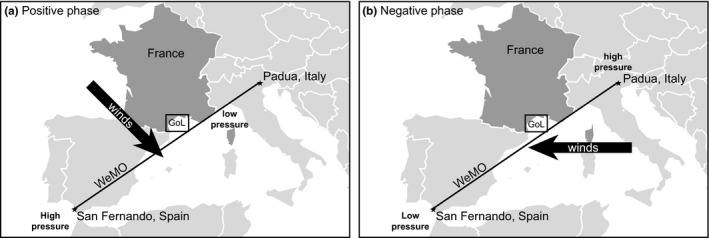
Patterns of WeMO influence over the northwestern Mediterranean Sea during its (a) positive and (b) negative phases (modified from http://www.ub.edu/gc/English/wemo.htm). GoL, Gulf of Lions

The WeMO is a low‐frequency variability pattern of atmospheric circulation that was first described by Martin‐Vide and Lopez‐Bustins (2006). Its index (hereafter, WeMO index) corresponds to the difference in standardized surface atmospheric pressures in San Fernando (Spain) and Padua (Italy; Figure 2). The north of Italy is subjected to relatively high barometric variability owing to the influence of the central European anticyclone and the Ligurian low‐pressure area, while southwestern Spain is frequently subjected to the influence of the Azores anticyclone. The transect linking these two zones covers the NW Mediterranean Sea. During the positive phase (Figure 2a), the anticyclone over the Azores encloses the southwest of Spain and the low pressures in the Ligurian Gulf result in winds blowing from the NW. During the negative phase (Figure 2b), the central European anticyclone located north of Italy and a low‐pressure center, in the Iberian SW, results in winds blowing from the east. In the Bay of Banyuls‐sur‐Mer, the negative phase is therefore associated with easterlies, which lead to frequent resuspension events. We used WeMO index data from http://www.ub.edu/gc/English/wemo.htm.

For the NAO and WeMO indices, the winter value for year *n* corresponds to an average from December year *n*‐1 to February year *n*. Annual and spring values corresponded to the average of monthly values from January to December and from March to May, respectively.

### 2.5 Environmental parameters

Water flow of the Rhône River was provided by Banque Hydro (http://www.hydro.eaufrance.fr). Air temperature, precipitation, wind speed, and Sea Level Pressure (SLP) were measured daily at Cap Béar station by Météo‐France (Figure 1b). We used the monthly averaged data available at https://donneespubliques.meteofrance.fr. Suspended Particulate Matter concentrations (SPM) were measured weekly 5 m above the bottom of the SOLA station (Service d'Observation du Laboratoire Arago, Bay of Banyuls‐sur‐Mer, 27 m depth; Figure 1b) within the framework of the Service d'Observation en Milieu Littoral (http://somlit.epoc.u-bordeaux1.fr/fr). Criteria 2 (C2) proposed by Labrune, Grémare, Guizien, et al. (2007) was used as a proxy for intense resuspension events. In brief, C2 corresponds to an estimated number of resuspension events per year. An intense resuspension event was assumed to take place during each day featuring both a wind direction between 90° and 170° and a decrease in SLP higher than 5 hPa either between (a) the day before and the day of measurement or (b) the day of measurement and the day after. For all parameters, seasonal values were computed as described above for NAO and WeMO indices.

### 2.6 Data analysis

#### 2.6.1 Grain‐size analysis

Hierarchical cluster analysis (normalized data, Euclidean distance, group average linking) was performed on site grain‐size fractions (expressed as percentage: <30, 30–63, 63–250, 250–500, 500–2,000 µm).

#### 2.6.2 Benthic macrofauna

To enable better community descriptions and allow comparison with previous studies in the area, data from replicate grabs per sampling site were pooled (Ellingsen, 2001). The results of data analyzed by averaging replicates per site are available in Supplementary material (i.e., Tables S1–S4 corresponding to Tables 3, 4, 5, 6; and Figures S1–S3 corresponding to Figures 5, 6, 7). When possible, taxa were identified to species level, but taken to a higher taxonomic level when confidence was low, thereby allowing species data to be comparable across datasets (i.e., from different years). Synonyms of scientific names of species were updated using the World Register of Marine Species (WoRMS Editorial Board, 2018). Species richness (taxa.0.5 m^−2^), total abundance (ind.0.5 m^−2^), and biomass (mgAFDW.0.5 m^−2^; ash‐free dry weight) were used as global descriptors of benthic macrofauna composition. Abundance‐based compositions were visualized using nMDS and hierarchical cluster analysis (square root‐transformed data, Bray–Curtis similarity, group average linking). We tested whether multivariate within‐group dispersion was homogenous for sampling site groups using the PERMDISP procedure (Anderson, 2001, 2006). SIMilarity PERcentage (SIMPER) analysis (Clarke, Somerfield, & Gorley, 2008) was performed to identify the species contributing most to dissimilarity between subclusters.

#### 2.6.3 Relationships linking climatic variability, environmental parameters, and benthic macrofauna

All analyses relating climatic variability and environmental parameters were run using benthic macrofauna by site rather than the clusters identified by the hierarchical cluster analysis because it is likely that environmental forcing is different at each depth. Spearman correlation was first used to assess the relationships between (annual, spring, and winter) NAO and WeMO indices, and the (a) main environmental parameters (see Section 2.5), (b) *D*_0.5_ and percentage of fine sediment (% <63 µm), (c) global descriptors of benthic macrofauna (species richness, abundance, and biomass), and (d) the abundances of the five species contributing most to temporal dissimilarities in benthic macrofauna composition within the three main clusters resulting from the hierarchical analysis. The Mantel test was used to assess the significance of correlations between the similarity matrices based on NAO and WeMO indices, and the similarity matrices based on (a) main environmental parameters, (b) sediment grain‐size fractions, and (c) the abundances of benthic macrofauna. A *BEST* procedure (Clarke & Ainsworth, 1993) was performed (999 permutations) to identify the subset of variables that best described temporal changes in benthic macrofauna composition at each sampling site. The global set of tested variables included WeMO index, NAO index, Suspended Particulate Matter (SPM), precipitation, air temperature, wind speed, Sea Level Pressure (SLP), Rhône River water flow, criteria 2 (C2), and sediment grain‐size. Some of these parameters were excluded to avoid collinearity, and this procedure was carried out for each integration period separately (whole year, springtime, and wintertime). With the exception of Spearman linear correlation (computed with R language; R Core Team, 2014), all analyses were completed using the PRIMER 6^®^ software package (Clarke & Gorley, 2006; Clarke & Warwick, 2001).

## 3 RESULTS

### 3.1 Temporal changes in climatic indices and environmental parameters

Strong temporal changes in annual, spring, and winter values of the NAO index were recorded between 2004 and 2013. The annual NAO index was between −0.76 (2005) and 1.07 (2011) except in 2010, when it was extremely low (−3.62). In spring, the NAO index presented an alternation of phases with a period close to 2 years. The winter NAO index values were slightly higher to those of the annual NAO index during most of the observed period. Strong temporal changes in annual, spring, and winter WeMO index values were also observed between 2004 and 2013. The annual WeMO index was negative and tended to be constant around −0.09, except in 2011 (−0.92). In spring, the WeMO index presented values close to the annual ones except in 2011. In winter, the WeMO index showed a decreasing trend between 2004 and 2008 and an increasing one between 2010 and 2012–2013.

Temporal changes in the main assessed environmental parameters are not shown, but their correlation with the values of both the NAO and the WeMO indices over (a) an annual period, (b) springtime, and (c) wintertime is shown in Table 2. Both NAO and WeMO indices correlated significantly with more environmental parameters during wintertime than during springtime and the whole year. There was a significant correlation between winter NAO index values and precipitation, Rhône River flow, and SLP; and winter WeMO index values and precipitation, wind speed, and Rhône River Water flow.

**Table 2:** Correlation coefficients of Spearman (*ρ*) between climatic indices and environmental parameters for each integration period separately

Environmental parameters	NAO index			WeMO index		
	Annual	Spring	Winter	Annual	Spring	Winter
Air temperature	0.37** *N* = 94	0.29* *N* = 54	0.25 *N* = 53	−0.40*** *N* = 54	−0.22 *N* = 54	0.04 *N* = 53
SLP	0.55*** *N* = 54	0.48 *** *N* = 54	0.87*** *N* = 53	−0.09 *N* = 54	−0.10 *N* = 54	−0.18 *N* = 53
Wind speed	−0.13 *N* = 54	−0.04 *N* = 54	0.09 *N* = 53	0.02 *N* = 54	0.28* *N* = 54	0.39** *N* = 53
Precipitation	−0.06 *N* = 54	−0.22 *N* = 54	−0.28* *N* = 53	−0.04 *N* = 54	−0.20 *N* = 54	−0.36** *N* = 53
Rhône River water flow	−0.37*** *N* = 94	−0.39*** *N* = 94	−0.34*** *N* = 93	0.47*** *N* = 94	0.54*** *N* = 94	0.40*** *N* = 93
SPM	−0.46 *N* = 12	0.01 *N* = 11	−0.28 *N* = 11	−0.10 *N* = 12	0.01 *N* = 11	0.44 *N* = 11
Criteria 2	0.16 *N* = 49	0.09 *N* = 48	−0.25 *N* = 47	0.20 *N* = 49	−0.03 *N* = 48	−0.03 *N* = 47

Abbreviations: NAO, North Atlantic Oscillation; SLP, Sea Level Pressure; SPM, Suspended Particulate Matter; WeMO, Western Mediterranean Oscillation.

*** *p* < .001,

** *p* < .01,

* *p* < .05.

### 3.2 Temporal changes in sediment grain‐size

**Figure 3:**
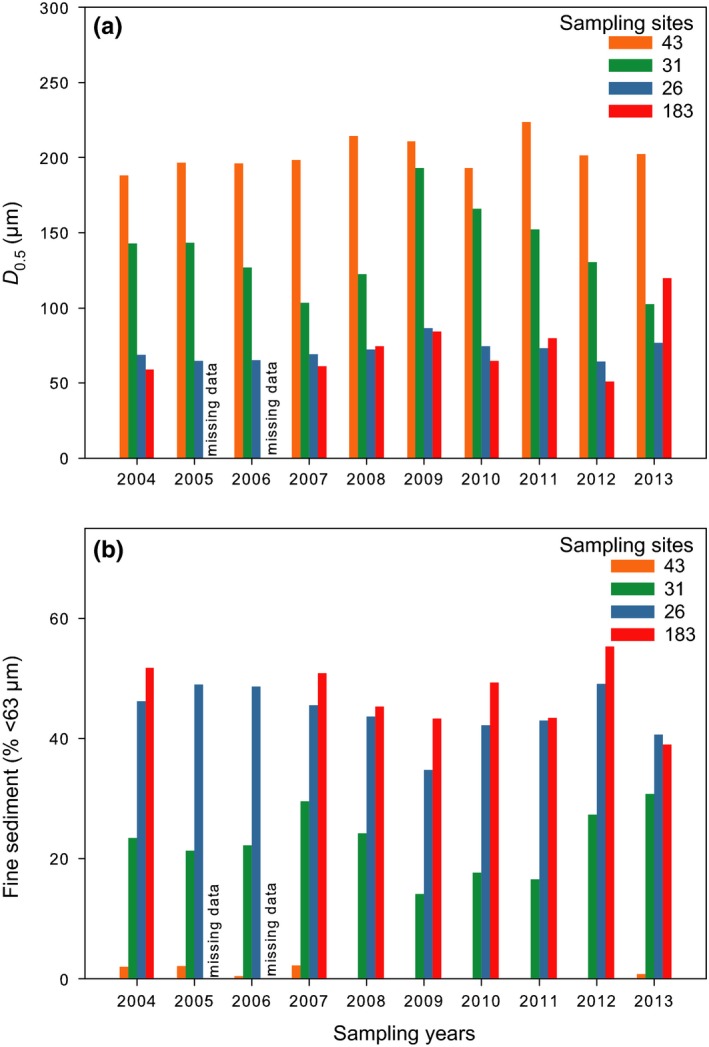
Temporal changes in (a) *D*_0.5_ (µm) and (b) fine sediment (% <63 µm)

Temporal changes in *D*_0.5_ were limited at sampling sites 43 and 26, high at sampling site 31, and intermediate at sampling site 183 (Table 3 and Figure 3a). Sediment at sampling site 43 was composed of well‐sorted fine sands with *D*_0.5_ between 188 (2004) and 223 µm (2011). Sediment at sampling site 31 was composed of muddy sands with *D*_0.5_ between 102 (2013) and 193 µm (2009). Sediment at sampling site 26 was composed of sandy mud with *D*_0.5_ between 64 (2012) and 86 µm (2009). Sediment at sampling site 183 was composed of mud with *D*_0.5_ between 59 µm (2004) and 120 µm (2013). Temporal changes in the percentage of fine sediment were low at sampling site 43, high at sampling site 31, and intermediate at sampling sites 26 and 183 (Table 3 and Figure 3b). At sampling site 43, fine sediment was absent between 2008 and 2012. Otherwise, their proportion was between 0.5 (2006) and 2.2% (2005, 2007). At sampling site 31, the proportion of fine sediment was between 14.1 (2009) and 30.8% (2013). At sampling site 26, a clear decreasing trend in the proportion of fine sediment was observed between 2005 (49.0%) and 2009 (34.8%). This was followed by an increasing trend up to 40.1% in 2012. At sampling site 183, the proportion of fine sediment was between 39.0 (2013) and 55.3% (2012). There was no significant correlation between NAO and WeMO indices, and *D*_0.5_ or the proportion of fine sediment at any sampling site (data not shown, *p* > .05 in all cases).

**Table 3:** Global descriptors of sediment grain‐size (*D*_0.5_ in µm and proportion of fine sediment in % <63 µm) and benthic macrofauna composition (species richness in taxa.0.5 m^−2^, abundance in ind.0.5 m^−2^, and biomass in mgAFDW.0.5 m^−2^)

Sampling site	Descriptor	2004	2005	2006	2007	2008	2009	2010	2011	2012	2013
43	*D*_0.5_	188.2	196.5	196.0	198.4	214.4	210.9	193.0	223.6	201.6	202.4
	Fine sediment	2.1	2.2	0.5	2.2	0.0	0.0	0.0	0.0	0.0	0.8
	Species richness	38	26	25	58	29	16	32	45	49	43
	Abundance	208	613	113	251	73	41	70	97	141	355
	Biomass	0.42	0.97	0.12	0.49	0.58	0.04	0.04	0.38	0.14	0.54
31	*D*_0.5_	142.8	143.5	126.8	103.2	122.6	193.0	166.0	152.3	130.6	102.7
	Fine sediment	23.4	21.4	22.2	29.5	24.2	14.1	17.7	16.5	27.3	30.8
	Species richness	56	54	30	52	34	46	41	45	64	105
	Abundance	437	222	153	378	132	516	401	529	500	796
	Biomass	0.47	0.37	0.16	0.28	0.18	0.52	0.39	0.44	0.54	2.09
26	*D*_0.5_	68.6	64.5	64.9	69.3	72.1	86.6	74.4	73.3	64.3	76.7
	Fine sediment	46.2	49.0	48.6	45.5	43.7	34.8	42.2	43.0	49.1	40.6
	Species richness	63	66	55	72	48	48	93	71	108	98
	Abundance	512	443	363	396	171	235	509	658	998	837
	Biomass	1.12	3.44	2.14	0.79	0.29	0.36	1.21	0.91	1.03	3.10
183	*D*_0.5_	59.0	NA	NA	61.1	74.5	84.1	64.6	79.7	51.0	120.0
	Fine sediment	51.7	NA	NA	50.8	45.3	43.3	49.3	43.4	55.3	39.0
	Species richness	83	62	53	72	55	54	101	90	110	83
	Abundance	455	425	186	387	275	208	602	576	723	446
	Biomass	1.82	2.70	1.24	5.57	1.79	2.90	2.63	3.98	4.64	2.27

Abbreviation: *D*_0.5_: median grain diameter.

**Figure 4:**
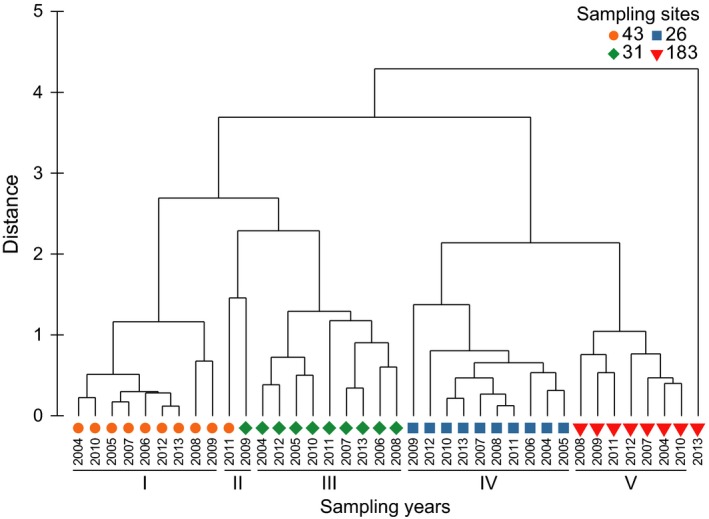
Hierarchical cluster analysis (Euclidean distance and average group method) of normalized grain‐size fractions (<30, 30–63, 63–250, 250–500, 500–2,000 µm). Symbols indicate sampling sites: 43 (circle), 31 (diamond), 26 (square), and 183 (triangle)

The hierarchical cluster analysis of normalized sediment grain‐size data (Figure 4) identified five main clusters (at 2.1% dissimilarity level) and showed the occurrence of important temporal changes within each community associated with the clusters. Cluster I corresponded mostly to sampling site 43 with years 2004–2010, 2012, and 2013. Cluster II corresponded to sampling site 43 (2011) and sampling site 31 (2009). Cluster III corresponded mostly to sampling site 31 with years 2004–2008 and 2010–2013. Cluster IV corresponded to sampling site 26 with all years. Cluster V corresponded mostly to sampling site 183 with years 2004–2012. One sample did not group with any cluster (sampling site 183 with year 2013). The only significant correlation between climatic indices and sediment grain‐size was observed at sampling site 43 during springtime for WeMO (Mantel test, *ρ* = .44, *p* < .05).

### 3.3 Temporal changes in benthic macrofauna

**Figure 5:**
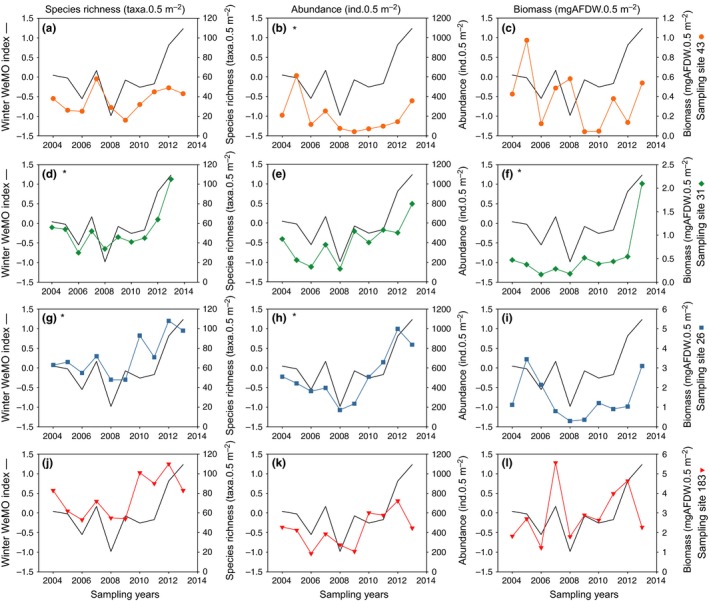
Temporal changes in the winter WeMO index and benthic macrofauna species richness, abundance, and biomass for each sampling site: (a, b, c) sampling site 43, (d, e, f) sampling site 31, (g, h, i) sampling site 26, and (j, k, l) sampling site 183. Symbols indicate sampling sites: 43 (circle), 31 (diamond), 26 (square), and 183 (triangle). *Significant (*p* < .05) linear correlation

In total, 15,431 specimens belonging to 448 taxa were identified. An overarching pattern was observed for temporal changes in macrobenthos species richness and abundance (Table 3 and Figure 5), which consisted of (a) a decreasing trend from 2004 to 2008/2009 with a higher value in 2007 at all sampling sites and (b) an increasing trend from 2010 to 2013 occasionally associated with lower values in 2011 and 2013 at deeper sampling sites (species richness at sampling site 26; both species richness and abundance at sampling site 183). Conversely, changes in biomass did not show any clear temporal pattern.

**Figure 6:**
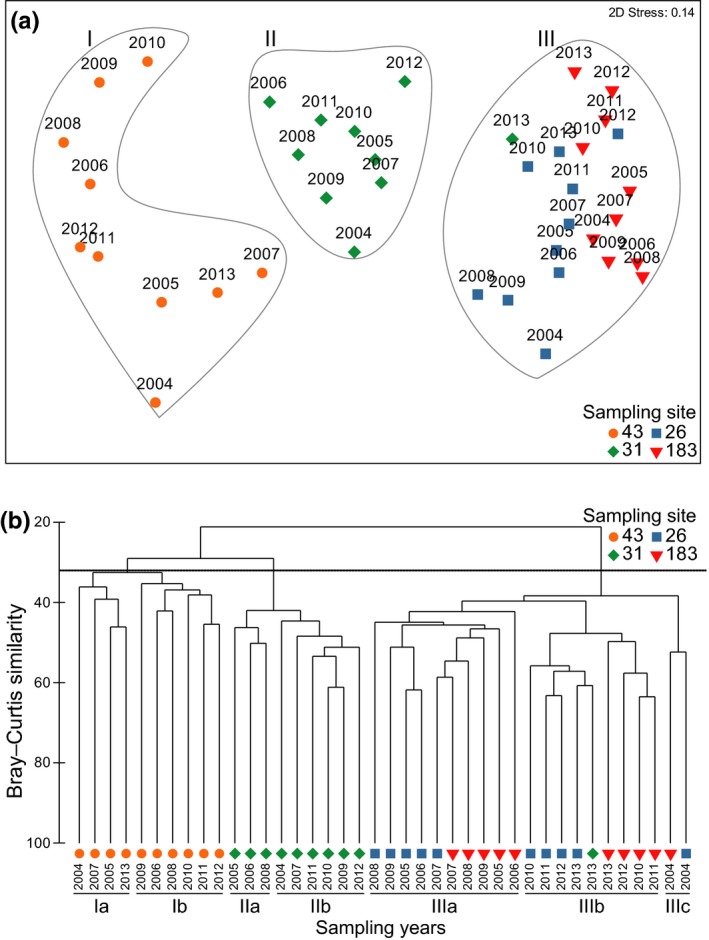
(a) Nonmetric multidimensional scaling (nMDS) and (b) hierarchical cluster analysis (square‐root‐transformed data, Bray–Curtis similarity, and average group method) of macrofauna species abundance data. Symbols indicate sampling sites: 43 (circle), 31 (diamond), 26 (square), and 183 (triangle)

The nMDS (Figure 6a) showed that temporal changes in benthic macrofauna composition were most important at sampling site 43. This was confirmed by the comparison of the dispersions of sites; pairwise comparison showed that for site 43, mean average dispersion was 43.68 ± 1.16 (standard error) and was significantly higher than dispersion values at all others sites (sampling site 31 with 37.77 ± 1.51; 26 with 36.12 ± 1.63; and 183 with 36.93 ± 1.20; *p* ≤ .01). The hierarchical clustering (Figure 6b) showed the existence of three main clusters at 32% similarity level: (I) all sampling years of sampling site 43; (II) all sampling years of sampling site 31, except 2013; and (III) all sampling years of sampling sites 26 and 183 and 2013 of sampling site 31. Clusters I and II could be subdivided into two subclusters: (Ia) years 2004, 2005, 2007, and 2013; (Ib) years 2006, 2008, and 2010–2012; (IIa) years 2005, 2006, and 2008; and (IIb) years 2004, 2007, and 2009–2012. Cluster III could be subdivided into three subclusters: (IIIa) 2005–2009 of sampling sites 26 and 183; (IIIb) years 2010–2013 of sampling sites 26 and 183 plus the year 2013 of sampling site 31; and (IIIc) year 2004 of sampling sites 26 and 183.

The species most responsible for dissimilarity between subcluster located at shallower waters were *Ditrupa arietina* and *Aspidosiphon muelleri* (clusters I and II), whereas those responsible for dissimilarity between subcluster deeper water (cluster III) were *A. muelleri* and *Turritella communis* (Table 4).

**Table 4:** Abundance, contribution, and cumulative contributions to dissimilarities in benthic macrofauna composition for the five species most responsible for dissimilarity between the subclusters identified in Figure 6b

Subclusters	Species	Average abundance	Average abundance	Contribution (%)	Cumulated (%)
		Ia	Ib		
Ia and Ib	*Ditrupa arietina*	243.8	21.8	55.6	55.6
	*Siphonoecetes neapolitanus*	6.5	8.3	2.9	58.5
	*Apseudopsis* spp.	7.3	0.3	2.4	60.9
	*Urothoe grimaldii*	5.8	1.3	2.1	62.9
	*Urothoe hesperia*	8.0	6.8	1.7	64.6
		IIa	IIb		
IIa and IIb	*Aspidosiphon muelleri*	43.0	160.2	28.2	28.2
	*Ditrupa arietina*	15.3	117.0	23.9	52.1
	*Owenia fusiformis*	8.0	19.8	3.7	55.8
	*Anapagurus breviaculeatus*	17.0	7.5	3.2	59.1
	*Turritella communis*	1.0	11.8	2.9	61.9
		IIIa	IIIb		
IIIa and IIIb	*Aspidosiphon muelleri*	29.2	119.6	15.6	15.6
	*Turritella communis*	10.0	62.7	7.7	23.4
	*Galathowenia oculata*	4.2	53.1	7.2	30.5
	*Nephtys kersivalensis*	27.5	50.6	6.0	36.5
	*Apseudopsis* spp.	35.9	15.4	5.4	41.9
		IIIa	IIIc		
IIIa and IIIc	*Turritella communis*	10.0	80.5	13.7	13.7
	*Apseudopsis* spp.	35.9	43.5	8.2	21.9
	*Lumbrineris latreilli*	29.2	18.5	5.0	26.9
	*Aspidosiphon muelleri*	29.2	11.5	4.5	31.4
	*Nephtys kersivalensis*	27.5	27.0	4.0	35.4
		IIIb	IIIc		
IIIb and IIIc	*Aspidosiphon muelleri*	119.5	11.5	14.1	14.1
	*Turritella communis*	62.7	80.5	7.2	21.4
	*Galathowenia oculata*	53.1	3.0	6.0	27.4
	*Apseudopsis* spp.	15.4	43.5	4.9	32.3
	*Nephtys kersivalensis*	50.6	27.0	4.6	36.9

### 3.4 Relationship between climatic indices, environmental parameters, sediment grain‐size, and benthic macrofauna

No significant correlation between the annual or winter NAO index values and the global descriptor of benthic macrofauna was found for any sampling site (Table 5). Conversely, the spring NAO index values correlated significantly with benthic macrofauna biomass at sampling site 26. No significant correlation was found between the annual or spring WeMO index values and the global benthic macrofauna descriptor for sampling sites (Table 5). Conversely, the winter WeMO values correlated significantly with benthic macrofauna descriptors: abundance at sampling site 43; species richness and biomass at sampling sites 31; and species richness and abundance at sampling site 26 (Table 5). Moreover, the general pattern of temporal changes in species richness and abundances observed at all sampling sites matched well with the consecutive decreasing and increasing trends recorded for the winter WeMO values. The peak in species richness and abundances observed at all four sampling sites in 2007 seemed linked to high winter WeMO values, whereas the low species richness and abundances recorded in 2008 tended to be associated with strongly negative values of the WeMO index (Table 3 and Figure 5). The only significant correlations between similarity matrices based on climatic indices and (a) benthic macrofauna species abundance and (b) biomass groups were observed at sampling site 31 for winter WeMO values (Mantel tests, *ρ* = .47 and *ρ* = .61, respectively, *p* < .01 in both cases).

**Table 5:** Correlation coefficients of Spearman (*ρ*) between climatic indices and global descriptors of benthic macrofauna (species richness in taxa.0.5 m^−2^, abundance in ind.0.5 m^−2^, and biomass in mgAFDW.0.5 m^−2^)

Sampling site	Benthic macrofauna	NAO index			WeMO index		
		Annual	Spring	Winter	Annual	Spring	Winter
43	Species richness	0.55	0.33	0.04	0.02	0.38	0.59
	Abundance	0.18	−0.33	0.22	−0.01	0.18	0.66*
	Biomass	0.31	−0.30	0.38	−0.13	0.09	0.21
31	Species richness	0.03	−0.14	0.22	0.58	0.49	0.95***
	Abundance	0.14	0.19	−0.39	0.38	0.15	0.59
	Biomass	−0.09	−0.02	−0.09	0.62	0.38	0.71*
26	Species richness	−0.07	−0.21	−0.15	−0.01	0.32	0.66*
	Abundance	0.09	−0.09	−0.21	0.05	0.15	0.67*
	Biomass	−0.41	−0.73*	−0.32	−0.25	−0.26	0.27
183	Species richness	0.03	0.05	−0.19	0.01	0.10	0.44
	Abundance	−0.04	0.01	−0.16	−0.02	0.01	0.39
	Biomass	0.10	0.53	0.21	0.09	0.16	0.50

Abbreviations: NAO, North Atlantic Oscillation; WeMO, Western Mediterranean Oscillation.

*** *p* < .001,

** *p* < .01,

* *p* < .05.

**Figure 7:**
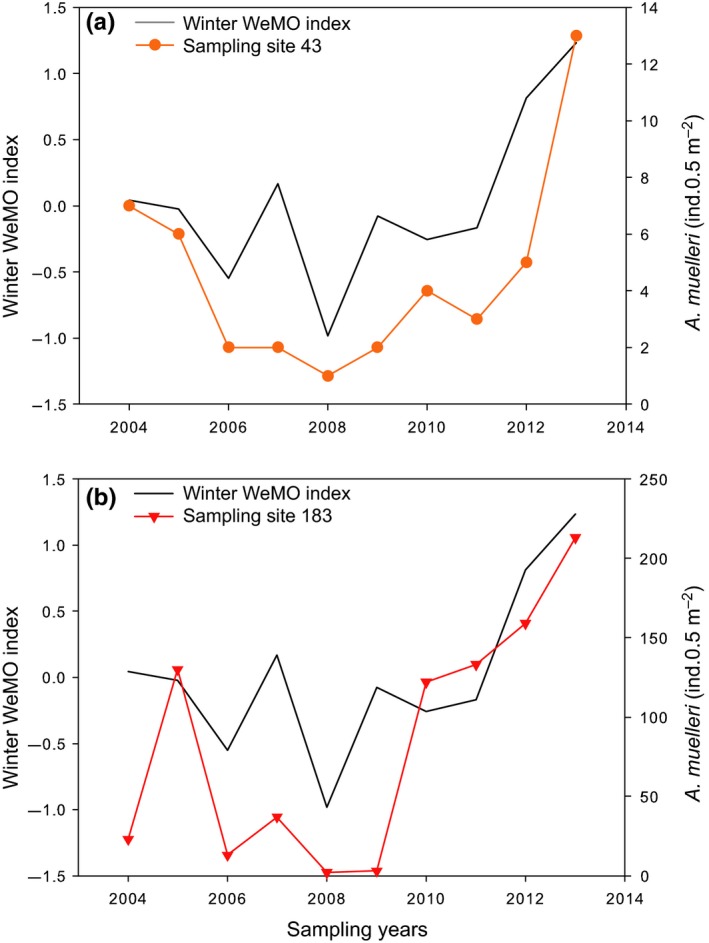
Temporal changes in winter WeMO index and abundance of *Aspidosiphon muelleri* at sampling sites (a) 43 and (b) 183. Correlations were significant in all cases. Symbols indicate sampling sites: 43 (circle) and 183 (triangle)

There was no significant correlation between NAO index values and the abundance of species contributing most to dissimilarities between subclusters at all four sampled sites. The only significant (*p* < .05 in all cases) correlations between WeMO values and the abundances of these species were recorded for wintertime WeMO and with (a) *Aspidosiphon muelleri* at sampling sites 43 (*ρ* = .68) and 183 (*ρ* = .67; Figure 7); (b) *Nephtys kersivalensis* at sampling site 31 (*ρ* = .67); and (c) *Urothoe hesperia* at sampling site 43 (*ρ* = .65).

For sampling sites 31, 26, and 183, neither *D*_0.5_ nor the proportion of fine sediment correlated with any global descriptor of benthic macrofauna. The only significant correlation was reported for sampling site 43 between the proportion of fine sediment and benthic macrofauna abundance (*ρ* = .73, *p* < .05). The only significant correlation between similarity matrices based on sediment grain‐size composition and benthic macrofauna composition was observed at sampling site 183 for biomass (Mantel test, *ρ* = .64, *p* < .05).

When computed on an annual basis, no set of variables could account for temporal changes in benthic macrofauna abundance and biomass at any sampling site (Table 6). This was also the case for benthic macrofauna abundance with springtime climatic indices, environmental parameters, and grain‐size except for macrofauna biomass at sampling site 26, which correlated with the combination of air temperature, precipitation, wind speed, and SPM. When computed during winter, subsets of climatic indices, environmental parameters, and grain‐size correlated significantly with changes in benthic macrofauna abundance at sampling sites 43, 31, 26, and 183 and with biomass at sampling site 31 (Table 6). The corresponding sets of contributing variables included (a) precipitation and fine sediment (abundance, sampling site 43, *p* = .04); (b) WeMO index, precipitation, and Rhône River water flow (abundance, sampling site 31); (c) WeMO index, precipitation, and C2 (abundance, sampling site 26); (d) precipitation and Rhône River water flow (abundance, sampling site 183); and (e) WeMO index, precipitation, wind speed, Rhône River water flow, and SPM (biomass, sampling site 31).

**Table 6:** Results from the *Best* procedure used to link benthic macrofauna abundance and biomass composition with climatic indices (computed for each integration period separately), and environmental and granulometric parameters

Abundance	Annual				Spring				Winter			
Sampling site	43	31	26	183	43	31	26	183	43	31	26	183
NAO index					X	X		X		X		X
WeMO index												
Air temperature												
SLP							X		X		X	
Wind speed					X	X		X				
Precipitation												
Rhône River water flow							X					
SPM												
Criteria 2												
*D*_0.5_		X	X	—		X		—		X	X	—
Fine sediment (%<63 µm)				—			X	—				—
*ρ*	0.40	0.36	−0.04	0.07	0.48	0.35	0.43	0.34	0.54*	0.68**	0.66**	0.56*

Biomass	Annual				Spring				Winter			
Sampling site	43	31	26	183	43	31	26	183	43	31	26	183
NAO index					X	X				X	X	X
WeMO index												
Air temperature												
SLP							X	X	X			
Wind speed					X	X						
Precipitation												
Rhône River water flow							X	X				
SPM												
Criteria 2												
*D*_0.5_		X		—		X		—		X	X	—
Fine sediment (%<63 µm)			X	—			X	—				—
*ρ*	0.52	0.49	0.23	0.04	0.27	0.38	0.60*	0.28	0.30	0.72**	0.32	0.32

Note

Black cells correspond to the parameters retained in the final model. Cells with an “X” correspond to the parameters which were not included in the model due to collinearity. Cells with “—” correspond to the parameters which were not included because of missing data for 2005 and 2006.

Abbreviations: *D*_0.5_, median grain diameter; NAO, North Atlantic Oscillation; SLP, Sea Level Pressure; SPM, Suspended Particulate Matter; WeMO, Western Mediterranean Oscillation.

*** *p* < .001,

** *p* < .01,

* *p* < .05.

The results of data analyzed by pooling replicates per site were the same as averaging replicates per site (Supplementary material), except for the subset of variables related to abundance at site 43 during winter (*p* = .05).

## 4 DISCUSSION

Previous studies have shown major temporal changes in the benthic macrofauna composition in the Gulf of Lions, based on (a) the initial observation of the boom of populations of the polychaete *Ditrupa arietina* in the Bay of Banyuls‐sur‐Mer (Grémare, Sardá, et al., 1998), (b) long‐term comparisons of benthic fauna composition in the same bay and later at the scale of the whole Gulf of Lions (Bonifácio et al., 2018; Grémare, Amouroux, & Vétion, 1998), and (c) data compilation regarding the dynamics of *D. arietina* along the Gulf of Lions (Bonifácio et al., 2018; Grémare, Sardá, et al., 1998). Based on Medernach et al. (2000) earlier detailed study of population dynamics of *D. arietina* and morphological characteristics of its early benthic stages (i.e., positive buoyancy before tube calcification), Labrune, Grémare, Guizien, et al. (2007) proposed that the abundance of this species and, thus, the composition of shallow communities could be influenced by the occurrence of resuspension events occurring during its recruitment period (e.g., springtime). Bonifácio et al. (2018) further tested this hypothesis by carrying out a long‐term comparison of benthic macrofauna composition in the whole Gulf of Lions between 1998 and 2010. In addition, they compiled annual NAO and WeMO indices versus *D. arietina* abundance data (at scale of the whole Gulf of Lions) between 1989 and 2013. Overall, their results reinforced the hypothesis put forward by Labrune, Grémare, Guizien, et al. (2007) and the hypothesized role of NAO in controlling benthic macrofauna composition in the Gulf of Lions.

All these studies, however, were based on either long‐term comparison data collected during specific time periods with long intervals (Bonifácio et al., 2018; Grémare, Amouroux, & Vétion, 1998; Labrune, Grémare, Guizien, et al., 2007) or on indicator species (Grémare, Sardá, et al., 1998; Medernach et al., 2000), which clearly complicates ecological interpretations and reduces the strength of derived conclusions (Grémare, Amouroux, & Vétion, 1998; Pearson, Josefson, & Rosenberg, 1985; Rosenberg, Gray, Josefson, & Pearson, 1987) when compared to studies based on long time series and/or the analysis of whole community composition (Hewitt et al., 2016; Kröncke et al., 1998, 2011, 2001). Within this context, the present study consisted of the acquisition and analysis of long time series collected within the Bay of Banyuls‐sur‐Mer at four sampling sites (located between 15 and 43 meters depth), which are representative of the main benthic communities described in this area (Guille, 1970).

### 4.1 Temporal changes in benthic macrofauna composition and potential indicator species

Our results first confirm the occurrence of major temporal (i.e., interannual) changes in the composition of benthic macrofauna at the four studied sites and showed that recent changes were most significant at site 43 (15 m depth), which support previous observations by Labrune, Grémare, Guizien, et al. (2007). Conversely, they do not support the fact that these changes tend to be more important at site 31 (26 m depth) than at sites 26 (31 m depth) and 183 (43 m depth) in Labrune, Grémare, Guizien, et al. (2007). The nMDS suggests more similar trajectories at sampling sites 26 and 183 than at site 43 with an opposition phase from winter WeMO index in most sampling sites showing, for instance, the extremely negative years (2006 and 2008) in opposition to extremely positive years (2012 and 2013). However, the moderate stress value of the nMDS representation (0.14) leads to some uncertainty in this interpretation. Furthermore, our 10‐year time series allows the identification of the species most responsible for interannual differences in benthic macrofauna composition at each sampled site. For instance, *Ditrupa arietina* represented 90.5% of macrofauna abundance at site 43 in 2005 (1,226 ind.m^−2^). *Ditrupa arietina* has already been identified as an indicator of temporal changes at both sandy sites (i.e., 43 and 31) by previous studies (Bonifácio et al., 2018; Grémare, Sardá, et al., 1998; Labrune, Grémare, Guizien, et al., 2007), which is confirmed by the present study. Another potential indicator species at site 31, already identified by Labrune, Grémare, Guizien, et al. (2007), is the sipunculid *Aspidosiphon muelleri*. Our data also lead to the identification of *A. muelleri* and *Turritella communis* as potential indicator species of temporal changes at sites 31, 26, and 183. Moreover, *A. muelleri* could be an indicator of climate change as its abundance significantly correlates with winter WeMO. However, more data are needed to identify indicator species of climate changes. Our results are in partial agreement with the results of Labrune, Grémare, Guizien, et al. (2007) who identified *T. communis* and *A. muelleri* as being among the five species that contributed most to dissimilarities in the 1967/68/1994/2003 benthic macrofauna composition at sites 26 and 183, respectively. Ferrero‐Vincente, Marco‐Méndez, Loya‐Fernández, and Sánchez‐Lizaso (2013) showed that shelter availability can be a limiting factor in the distribution of this sipunculid and that these animals are observed inhabiting empty tubes of *D. arietina* and gastropod shells (Ferrero‐Vincente, Marco‐Méndez, Loya‐Fernández, & Sánchez‐Lizaso, 2014). During the present study, *A. muelleri* was found both in *D. arietina* tubes and in *T. communis* shells. It contributed to more than 10% of total abundance at sites 31 and 183. The smallest individuals were observed at site 31 (personal observation), probably due to the presence of *D. arietina* tubes, which juveniles favored (Ferrero‐Vincente et al., 2014), whereas larger individuals found at site 183 tended to inhabit the shells of *T. communis*. It is, therefore, likely that the information provided by these three species would prove largely redundant since *A. muelleri* tend to occupy the empty tubes of *D. arietina* and *T. communis* (Ferrero‐Vicente et al., 2013; Ferrero‐Vincente et al., 2014).

### 4.2 NAO and WeMO indices, integration periods

Two of the major results from the present study were the lack of (a) apparent cyclicality in the composition of benthic macrofauna (a period of 8 years would be expected in the case of a tight control by the NAO; Da Costa & Verdiere, 2002) and (b) correlation between annual NAO and either the global characteristics or the composition of benthic macrofauna at all four sampled sites. Both of these results are in disagreement with the relationship between NAO and changes in benthic macrofauna composition in the coastal zone of the Gulf of Lions suggested by previous studies (Bonifácio et al., 2018; Labrune, Grémare, Guizien, et al., 2007).

Regarding the first result, NAO and WeMO indices can both be seen as proxies for changes in environmental parameters (Drinkwater et al., 2003; Lockerbie, Coll, Shannon, & Jarre, 2017; Ottersen et al., 2001). The NAO, however, is referring explicitly to the North Atlantic, whereas WeMO has been specifically designed for the western Mediterranean (Martin‐Vide & Lopez‐Bustins, 2006). It is, therefore, not surprising that Martin‐Vide and Lopez‐Bustins (2006) demonstrated a better correlation of the WeMO index than that of the NAO index with precipitations on the eastern coastline of the Iberian Peninsula. Likewise, Martín et al. (2012) also showed a better correlation between the WeMO index and water flow of the Rhône River than the NAO index. Our results are fully consistent with these data since we also observed better correlations (higher absolute value of Spearman coefficient; Table 2): (a) between WeMO versus Rhône River water flow than between NAO versus Rhône River water flow, although positive with WeMO and negative with NAO; and (b) between winter WeMO versus precipitation than winter NAO versus precipitation. Furthermore, SPM only correlated with winter WeMO, which tends to support the use of WeMO rather than NAO as a climatic index in the NW Mediterranean. The relationship between precipitation and Rhône river water flow with WeMO seems linked to the locality where they were sampled, while the precipitation was sampled at Cap Béar (really close of Banyuls‐sur‐Mer, see Figure 1), the water flow of Rhône was measured close the mouth and it accounts for the entire hydrological basin (97,800 km^2^) which is submitted to strong climatic heterogeneity (Pont, Simonnet, & Walter, 2002).

Regarding the most appropriate integration period, it was first suggested that NAO has a stronger control on the climate of the Northern Hemisphere during wintertime. During this season, the magnitude and spatial coherence of atmospheric circulation variability as well as the influence of circulation changes and large‐scale precipitation are stronger (Osborn, 2006; Osborn, Conway, Hulme, Gregory, & Jones, 1999). Our results suggest that such a reinforcement of climatic forcing influences the WeMO in the same way as indicated by the fact that the number of environmental parameters, which correlate significantly with WeMO was highest during wintertime. There are also good ecological rationales to believe that seasonal integration periods are more appropriate than annual ones to correlate climatic indices and/or environmental parameters with biological data. For example, the negative effects of severe winter temperature on benthic macrofauna communities have been highlighted in the zone off the Island of Norderney (Kröncke et al., 1998, 2001; Kröncke, Reiss, & Dippner, 2013), in the German Bight (Neumann, Ehrich, & Kröncke, 2008; Shojaei et al., 2016), and in the Wadden Sea (Beukema, Essink, & Dekker, 2000). Although all global benthic macrofauna descriptors studied present the same temporal trend, significant correlations were only observed between winter WeMO and macrofauna descriptors at sampling sites 43 (abundance), 31 (species richness and biomass), and 26 (species richness and abundance). The reasons for an absence of such significant correlation at site 183 remain unclear, but it might be linked to hydro‐sedimentary processes. For instance, storm resuspension event effects are known to be less frequent below 35 m depth (25–35 m depth; Ferré et al., 2005). Moreover, it was not possible to define any subset of climatic indices, environmental parameters, and granulometric data integrated over a full year or over springtime significantly accounting for temporal changes in benthic macrofauna abundance at any sampling site, whereas such subsets could be identified at sampling sites 43, 31, 26, and 183 when climatic indices, environmental parameters, and grain‐size were integrated over wintertime. Overall, this supports the idea that wintertime constitutes a key period in controlling benthic macrofauna composition in the Gulf of Lions as well. Also, our data are in accordance with Hewitt et al. (2016) who observed changes occurring in all ecological levels (individual species to community level) as a response to climate changes. It should nevertheless be pointed out that our sampling took place during this season as well, which may partly account for this result although the integration period of both climatic indices and environmental parameters corresponded to the winter before sampling.

### 4.3 Key environmental parameters involved

In the North Sea, the key environmental parameter controlling benthic macrofauna composition is wintertime temperature (Beukema et al., 2000; Kröncke et al., 1998, 2013, 2001; Neumann et al., 2008; Shojaei et al., 2016). Our results suggest similar processes occurring in the NW Mediterranean under hydro‐sedimentary forcing. First, two of the potential indicator species identified during the present study (i.e., *Ditrupa arietina* and *Turritella communis*) are both suspension‐feeders. *Turritella communis* can remain buried in mud filtering for long period unless disturbed (Yonge, 1946). Yonge reported that *T. communis* is very sensitive to SPM and that it stops its inhalant current as soon as fine sediment enters in its mantle cavity. Consequently, the negative phase of wintertime WeMO index, related with a high frequency of resuspension events, may have a strong impact on the population dynamic of this particular species. Our results suggest that the mediators between winter WeMO and benthic macrofauna (based on abundances at sampling sites 31, 26, and 183 and biomasses at sampling site 31) were as follows: winter precipitation, winter Rhône River water flow, winter C2, winter SPM, and winter wind speed. This is in accordance with Lloret et al. (2001) who suggest a link between recruitment and local environmental conditions such as river discharge, wind speed, and direction and climatic oscillation. Interestingly, most of these parameters are linked with hydro‐sedimentary processes. Suspended Particulate Matter, C2, and wind speed are all directly or indirectly related to sediment resuspension within the Bay of Banyuls‐sur‐Mer (Ferré et al., 2005; Grémare et al., 2003; Grémare, Amouroux, Charles, et al., 1998; Labrune, Grémare, Guizien, et al., 2007), whereas the Rhône River is the main source of continental particles for the Gulf of Lions (Durrieu de Madron et al., 2000). The impact of the Rhône River on benthic macrofauna composition has already been described in the immediate vicinity of the Rhône River mouth (Bonifácio et al., 2014, 2018; Darnaude, Salen‐Picard, Polunin, & Harmelin‐Vivien, 2004; Salen‐Picard, Arlhac, & Alliot, 2003; Salen‐Picard et al., 2003). Labrune, Grémare, Amouroux, et al. (2007) also identified that the composition of the Littoral Sandy Mud community slightly differed in the NE and SW part of the Gulf of Lions and attributed this to the proximity of the Rhône River. Subsequently, based on the resampling of the same sites in 2010, Bonifácio et al. (2018) showed that the explicit modeling of the proximity of the Rhône River strongly increased the proportion of the explained variance of the composition of benthic macrofauna at the five sampled depths (i.e., 10, 20, 30, 40, and 50 m). Our data support these results and suggest that temporal changes in Rhône River water flow may influence benthic macrofauna composition over the whole Gulf of Lions.

Overall, our results confirm the occurrence of major temporal changes in the composition of macrobenthic communities within the Gulf of Lions. They also support the use of several indicator species (i.e., *Ditrupa arietina*, *Turritella communis*, and *Aspidosiphon muelleri*) as proxies for temporal changes. Our results are also in partial agreement with the current paradigm according to which changes in benthic macrofauna composition were determined by climatic drivers through environmental (hydro‐sedimentary) processes. Nevertheless, and because it is the first study to involve the acquisition of long‐term time series of benthic macrofauna in the Catalan Sea, our study allows the paradigm to be refined by concluding that (a) the WeMO appears to be more closely related than NAO to benthic macrofauna composition in the Bay of Banyuls‐sur‐Mer, (b) winter is a better integration period than spring or the whole year to be used as a proxy in community composition changes, and finally, (c) Rhône River water flow is probably involved in the control of benthic macrofauna composition in the whole Gulf of Lions.

## CONFLICT OF INTEREST

None declared.

## AUTHOR CONTRIBUTIONS

AG, CL, and JMA designed and conceptualized the study. AG, CL, JMA, and PB performed the sampling. CL, JMA, and PB processed the samples. AG, CL, and PB analyzed and interpreted the data. AG, CL, JMA, and PB wrote the manuscript.

## Data Availability

Data produced from the present study, such as abundance, biomass, and sediment grain‐size, will be available in the macrobenthic French database developed by the “Réseau des Stations et Observatoires Marins”—RESOMAR (http://resomar.cnrs.fr/).

## References

[ece35569-bib-0001] Aloisi, J. C. , Got, H. , & Monaco, A. (1973). Carte géologique du précontinent languedocien au 1/250000ième. Enschede, The Netherlands: International Institute for Aerial Survey and Earth Sciences (ITC).

[ece35569-bib-0002] Anderson, M. J. (2001). A new method for non‐parametric multivariate analysis of variance. Austral Ecology, 26, 32–46. 10.1111/j.1442-9993.2001.01070.pp.x

[ece35569-bib-0003] Anderson, M. J. (2006). Distance‐based tests for homogeneity of multivariate dispersions. Biometrics, 62, 245–253. 10.1111/j.1541-0420.2005.00440.x 16542252

[ece35569-bib-0004] Beukema, J. J. (1985). Growth and dynamics in populations of *Echinocardium cordatum* living in the North Sea off the Dutch North coast. Netherlands Journal of Sea Research, 19, 129–134. 10.1016/0077-7579(85)90017-1

[ece35569-bib-0005] Beukema, J. J. , Essink, K. , & Dekker, R. (2000). Long‐term observations on the dynamics of three species of polychaetes living on tidal flats of the Wadden Sea: The role of weather and predator‐prey interactions. Journal of Animal Ecology, 69, 31–44. 10.1046/j.1365-2656.2000.00368.x

[ece35569-bib-0006] Bonifácio, P. , Bourgeois, S. , Labrune, C. , Amouroux, J. M. , Escoubeyrou, K. , Buscail, R. , … Grémare, A. (2014). Spatiotemporal changes in surface sediment characteristics and benthic macrofauna composition off the Rhône River in relation to its hydrological regime. Estuarine, Coastal and Shelf Science, 151, 196–209. 10.1016/j.ecss.2014.10.011

[ece35569-bib-0007] Bonifácio, P. , Grémare, A. , Gauthier, O. , Romero‐Ramirez, A. , Bichon, S. , Amouroux, J. M. , & Labrune, C. (2018). Long‐term (1998 vs. 2010) large‐scale comparison of soft‐bottom benthic macrofauna composition in the Gulf of Lions, NW Mediterranean Sea. Journal of Sea Research, 131, 32–45. 10.1016/j.seares.2017.08.013

[ece35569-bib-0008] Clarke, K. R. , & Ainsworth, M. (1993). A method of linking multivariate community structure to environmental variables. Marine Ecology Progress Series, 92, 205–219. 10.3354/meps092205

[ece35569-bib-0009] Clarke, K. R. , & Gorley, R. N. (2006). PRIMER v6: User manual/tutorial. Plymouth, UK: PRIMER‐E.

[ece35569-bib-0010] Clarke, K. R. , Somerfield, P. J. , & Gorley, R. N. (2008). Testing of null hypotheses in exploratory community analyses: Similarity profiles and biota‐environment linkage. Journal of Experimental Marine Biology and Ecology, 366, 56–69. 10.1016/j.jembe.2008.07.009

[ece35569-bib-0011] Clarke, K. R. , & Warwick, R. M. (2001). Change in marine communities: An approach to statistical analysis and interpretation (2nd ed.). Plymouth, UK: PRIMER‐E.

[ece35569-bib-0012] Da Costa, E. D. , & Verdiere, C. (2002). The 7.7‐year North Atlantic oscillation. Quarterly Journal of the Royal Meteorological Society, 128, 797–817.

[ece35569-bib-0013] Darnaude, A. M. , Salen‐Picard, C. , Polunin, N. V. , & Harmelin‐Vivien, M. L. (2004). Trophodynamic linkage between river runoff and coastal fishery yield elucidated by stable isotope data in the Gulf of Lions (NW Mediterranean). Oecologia, 138, 325–332. 10.1007/s00442-003-1457-3 14689296

[ece35569-bib-0014] De Backer, A. , Van Hoey, G. , Coates, D. , Vanaverbeke, J. , & Hostens, K. (2014). Similar diversity‐disturbance responses to different physical impacts: Three cases of small‐scale biodiversity increase in the Belgian part of the North Sea. Marine Pollution Bulletin, 84, 251–262. 10.1016/j.marpolbul.2014.05.006 24889315

[ece35569-bib-0015] Drinkwater, K. F. , Belgrano, A. , Borja, A. , Conversi, A. , Edwards, M. , Greene, C. H. , … Walker, H. (2003). The response of marine ecosystems to climate variability associated with the North Atlantic oscillation In Hurrell J. W., Kushnir Y., Ottersen G., & Visbeck M. (Eds.), The North Atlantic oscillation: Climatic significance and environmental impact (p. 279). Washington, DC: American Geophysical Union.

[ece35569-bib-0016] Durrieu de madron, X. , Abassi, A. , Heussner, S. , Monaco, A. , Aloisi, J. C. , Radakovitch, O. , … Kerherve, P. (2000). Particulate matter and organic carbon budgets for the Gulf of Lions (NW Mediterranean). Oceanologica Acta, 23, 717–730. 10.1016/S0399-1784(00)00119-5

[ece35569-bib-0017] Ellingsen, K. E. (2001). Biodiversity of a continental shelf soft‐sediment macrobenthos community. Marine Ecology Progress Series, 218, 1–15. 10.3354/meps218001

[ece35569-bib-0018] Fernandez de Puelles, M. , Valencia, J. , & Vicente, L. (2004). Zooplankton variability and climatic anomalies from 1994 to 2001 in the Balearic Sea (Western Mediterranean). ICES Journal of Marine Science, 61, 492–500. 10.1016/j.icesjms.2004.03.026

[ece35569-bib-0019] Ferré, B. , Guizien, K. , Durrieu de Madron, X. , Palanques, A. , Guillén, J. , & Grémare, A. (2005). Fine‐grained sediment dynamics during a strong storm event in the inner‐shelf of the Gulf of Lion (NW Mediterranean). Continental Shelf Research, 25, 2410–2427. 10.1016/j.csr.2005.08.017

[ece35569-bib-0020] Ferrero‐Vicente, L. M. , Marco‐Méndez, C. , Loya‐Fernández, Á. , & Sánchez‐Lizaso, J. L. (2013). Limiting factors on the distribution of shell/tube‐dwelling sipunculans. Journal of Experimental Marine Biology and Ecology, 446, 345–354. 10.1016/j.jembe.2013.06.011

[ece35569-bib-0021] Ferrero‐Vicente, L. M. , Marco‐Méndez, C. , Loya‐Fernández, A. , & Sánchez‐Lizaso, J. L. (2014). Observations on the ecology and reproductive biology of the sipunculan worm *Aspidosiphon muelleri* in temperate waters. Journal of the Marine Biological Association of the United Kingdom, 94, 1629–1638.

[ece35569-bib-0022] Fromentin, J. M. , & Planque, B. (1996). *Calanus* and environment in the eastern North Atlantic. II. Influence of the North Atlantic Oscillation on *C*. f*inmarchicus* and *C*. *helgolandicus* . Marine Ecology Progress Series, 134, 111–118. 10.3354/meps134111

[ece35569-bib-0023] Grémare, A. , Amouroux, J.‐M. , Cauwet, G. , Charles, F. , Courties, C. , De Bovée, F. , … Zudaire, L. (2003). The effects of a strong winter storm on physical and biological variables at a shelf site in the Mediterranean. Oceanologica Acta, 26, 407–419. 10.1016/S0399-1784(03)00029-X

[ece35569-bib-0024] Grémare, A. , Amouroux, J.‐M. , Charles, F. , Medernach, L. , Jordana, E. , Nozais, C. , … Colomines, J.‐C. (1998). Temporal changes in the biochemical composition of particulate organic matter sedimentation in the Bay of Banyuls‐sur‐Mer. Oceanologica Acta, 21, 783–792. 10.1016/S0399-1784(99)80006-1

[ece35569-bib-0025] Grémare, A. , Amouroux, J. M. , & Vétion, G. (1998). Long‐term comparison of macrobenthos within the soft bottoms of the Bay of Banyuls‐sur‐Mer (northwestern Mediterranean Sea). Journal of Sea Research, 40, 281–302. 10.1016/S1385-1101(98)00032-X

[ece35569-bib-0026] Grémare, A. , Sardá, R. , Medernach, L. , Jordana, E. , Pinedo, S. , Amouroux, J. M. , … Charles, F. (1998). On the dramatic increase of *Ditrupa arietina* O. F. Müller (Annelida Polychaeta) along both the French and the Spanish Catalan Coasts. Estuarine, Coastal and Shelf Science, 47, 447–457.

[ece35569-bib-0027] Guille, A. (1970). Bionomie benthique du plateau continental de la côte catalane française. II – Les communautés de la macrofaune. Vie Et Milieu, 21, 149–280.

[ece35569-bib-0028] Hagberg, J. , & Tunberg, B. G. (2000). Studies on the covariation between physical factors and the long‐term variation of the marine soft bottom macrofauna in Western Sweden. Estuarine, Coastal and Shelf Science, 50, 373–385. 10.1006/ecss.1999.0578

[ece35569-bib-0029] Hewitt, J. E. , Ellis, J. I. , & Thrush, S. F. (2016). Multiple stressors, nonlinear effects and the implications of climate change impacts on marine coastal ecosystems. Global Change Biology, 22, 2665–2675. 10.1111/gcb.13176 26648483

[ece35569-bib-0030] Hurrell, J. W. (1995). Decadal trends in the north Atlantic oscillation: Regional temperatures and precipitation. Science, 269, 676–679. 10.1126/science.269.5224.676 17758812

[ece35569-bib-0031] Hurrell, J. W. , & Deser, C. (2009). North Atlantic climate variability: The role of the North Atlantic oscillation. Journal of Marine Systems, 78, 28–41. 10.1016/j.jmarsys.2008.11.026

[ece35569-bib-0032] Keller, S. , Valls, M. , Hidalgo, M. , & Quetglas, A. (2014). Influence of environmental parameters on the life‐history and population dynamics of cuttlefish *Sepia officinalis* in the western Mediterranean. Estuarine, Coastal and Shelf Science, 145, 31–40. 10.1016/j.ecss.2014.04.016

[ece35569-bib-0033] Kröncke, I. , Dippner, J. W. , Heyen, H. , & Zeiss, B. (1998). Long‐term changes in macrofaunal communities off Norderney (East Frisia, Germany) in relation to climate variability. Marine Ecology Progress Series, 167, 25–36. 10.3354/meps167025

[ece35569-bib-0034] Kröncke, I. , Reiss, H. , & Dippner, J. W. (2013). Effects of cold winters and regime shifts on macrofauna communities in shallow coastal regions. Estuarine, Coastal and Shelf Science, 119, 79–90. 10.1016/j.ecss.2012.12.024

[ece35569-bib-0035] Kröncke, I. , Reiss, H. , Eggleton, J. D. , Aldridge, J. , Bergman, M. J. N. , Cochrane, S. , … Rees, H. L. (2011). Changes in North Sea macrofauna communities and species distribution between 1986 and 2000. Estuarine, Coastal and Shelf Science, 94, 1–15. 10.1016/j.ecss.2011.04.008

[ece35569-bib-0036] Kröncke, I. , Zeiss, B. , & Rensing, C. (2001). Long‐term variability in macrofauna species composition off the Island of Norderney (East Frisia, Germany) in relation to changes in climatic and environmental conditions. Senckenbergiana Maritima, 31, 65–82. 10.1007/BF03042837

[ece35569-bib-0037] Labrune, C. , Grémare, A. , Amouroux, J. M. , Sardá, R. , Gil, J. , & Taboada, S. (2007). Assessment of soft‐bottom polychaete assemblages in the Gulf of Lions (NW Mediterranean) based on a mesoscale survey. Estuarine, Coastal and Shelf Science, 71, 133–147. 10.1016/j.ecss.2006.07.007

[ece35569-bib-0038] Labrune, C. , Grémare, A. , Amouroux, J. M. , Sardá, R. , Gil, J. , & Taboada, S. (2008). Structure and diversity of shallow soft‐bottom benthic macrofauna in the Gulf of Lions (NW Mediterranean). Helgoland Marine Research, 62, 201–214. 10.1007/s10152-008-0108-9

[ece35569-bib-0039] Labrune, C. , Grémare, A. , Guizien, K. , & Amouroux, J. M. (2007). Long‐term comparison of soft bottom macrobenthos in the Bay of Banyuls‐sur‐Mer (north‐western Mediterranean Sea): A reappraisal. Journal of Sea Research, 58, 125–143. 10.1016/j.seares.2007.02.006

[ece35569-bib-0040] Lloret, J. , Lleonart, J. , Sole, I. , & Fromentin, J. M. (2001). Fluctuations of landings and environmental conditions in the north‐western Mediterranean Sea. Fisheries Oceanography, 10, 33–50. 10.1046/j.1365-2419.2001.00151.x

[ece35569-bib-0041] Lockerbie, E. M. , Coll, M. , Shannon, L. J. , & Jarre, A. (2017). The use of indicators for decision support in northwestern Mediterranean Sea fisheries. Journal of Marine Systems, 174, 64–77. 10.1016/j.jmarsys.2017.04.003

[ece35569-bib-0042] Martín, P. , Sabatés, A. , Lloret, J. , & Martin‐Vide, J. (2012). Climate modulation of fish populations: The role of the Western Mediterranean Oscillation (WeMO) in sardine (*Sardina pilchardus*) and anchovy (*Engraulis encrasicolus*) production in the north‐western Mediterranean. Climatic Change, 110, 925–939. 10.1007/s10584-011-0091-z

[ece35569-bib-0043] Martin‐Vide, J. , & Lopez‐Bustins, J. A. (2006). The Western Mediterranean oscillation and rainfall in the Iberian Peninsula. International Journal of Climatology, 26, 1455–1475. 10.1002/joc.1388

[ece35569-bib-0044] Martin‐Vide, J. , Sanchez‐Lorenzo, A. , Lopez‐Bustins, J. A. , Cordobilla, M. J. , Garcia‐Manuel, A. , & Raso, J. M. (2008). Torrential rainfall in northeast of the Iberian Peninsula synoptic patterns and WeMO influence. Advances in Sciences and Research, 2, 99–105. 10.5194/asr-2-99-2008

[ece35569-bib-0045] Massé, H. L. (2000). Long‐term changes in sand‐bottom macrofauna along the coast of Provence (northwest Mediterranean Sea). Oceanologica Acta, 23, 229–242. 10.1016/S0399-1784(00)00124-9

[ece35569-bib-0046] Medernach, L. , Jordana, E. , Grémare, A. , Nozais, C. , Charles, F. , & Amouroux, J. M. (2000). Population dynamics, secondary production and calcification in a Mediterranean population of *Ditrupa arietina* (Annelida: Polychaeta). Marine Ecology Progress Series, 199, 171–184. 10.3354/meps199171

[ece35569-bib-0047] MSFD (2008). Directive 2008/56/EC of the European Parliament and of the Council of 17 June 2008 establishing a framework for Community action in the field of marine environmental policy (Marine Strategy Framework Directive). Official Journal of the European Union, L164, 19–40.

[ece35569-bib-0048] Muñoz‐Expósito, P. , Macías, D. , Ortíz de Urbina, J. M. , García‐Barcelona, S. , Gómez, M. J. , & Báez, J. C. (2017). North Atlantic oscillation affects the physical condition of migrating bullet tuna *Auxis rochei* (Risso, 1810) from the Western Mediterranean Sea. Fisheries Research, 194, 84–88. 10.1016/j.fishres.2017.05.016

[ece35569-bib-0049] Neumann, H. , Ehrich, S. , & Kröncke, I. (2008). Effects of cold winters and climate on the temporal variability of an epibenthic community in the German Bight. Climate Research, 37, 241–251. 10.3354/cr00769

[ece35569-bib-0050] Osborn, T. J. (2006). Recent variations in the winter North Atlantic oscillation. Weather, 61, 353–355. 10.1256/wea.190.06

[ece35569-bib-0051] Osborn, T. J. , Conway, D. , Hulme, M. , Gregory, J. M. , & Jones, P. (1999). Air flow influences on local climate: Observed and simulated mean relationships for the United Kingdom. Climate Research, 13, 173–191. 10.3354/cr013173

[ece35569-bib-0052] Ottersen, G. , Planque, B. , Belgrano, A. , Post, E. , Reid, P. , & Stenseth, N. (2001). Ecological effects of the North Atlantic oscillation. Oecologia, 128, 1–14. 10.1007/s004420100655 28547079

[ece35569-bib-0053] Pearson, T. H. , Josefson, A. B. , & Rosenberg, R. (1985). Petersen's benthic stations revisited. I. Is the Kattegatt becoming eutrophic? Journal of Experimental Marine Biology and Ecology, 92, 157–206. 10.1016/0022-0981(85)90094-2

[ece35569-bib-0054] Pont, D. , Simonnet, J. P. , & Walter, A. V. (2002). Medium‐term changes in suspended sediment delivery to the ocean: Consequences of catchment heterogeneity and river management (Rhône River, France). Estuarine, Coastal and Shelf Science, 54, 1–18. 10.1006/ecss.2001.0829

[ece35569-bib-0055] R Core Team (2014). R: A language and environment for statistical computing v3.3.3. Vienna, Austria: R Foundation for Statistical Computing [Computer Software]. Retrieved from http://www.R-project.org/

[ece35569-bib-0056] Rees, H. L. , Pendle, M. A. , Limpenny, C. E. , Mason, C. E. , Boyd, S. E. , Birchenough, S. , & Vivian, C. M. G. (2006). Benthic responses to organic enrichment and climatic events in the western North Sea. Journal of the Marine Biological Association of the United Kingdom, 86, 1–18. 10.1017/S002531540601280X

[ece35569-bib-0057] Rosenberg, R. , Gray, J. S. , Josefson, A. B. , & Pearson, T. H. (1987). Petersen's benthic stations revisited. II. Is the Oslofjord and eastern Skagerrak enriched? Journal of Experimental Marine Biology and Ecology, 105, 219–251. 10.1016/0022-0981(87)90174-2

[ece35569-bib-0058] Salen‐Picard, C. (1981). Évolution d'un peuplement de Vase Terrigène Côtière soumis à des rejets de dragages dans le golfe de Fos. Tethys, 10, 83–88.

[ece35569-bib-0059] Salen‐Picard, C. , Arlhac, D. , & Alliot, E. (2003). Responses of a Mediterranean soft bottom community to short‐term (1993–1996) hydrological changes in the Rhone river. Marine Environmental Research, 55, 409–427. 10.1016/S0141-1136(02)00307-0 12628194

[ece35569-bib-0061] Santojanni, A. , Arneri, E. , Bernardini, V. , Cingolani, N. , Di Marco, M. , & Russo, A. (2006). Effects of environmental variables on recruitment of anchovy in the Adriatic Sea. Climate Research, 31, 181–193. 10.3354/cr031181

[ece35569-bib-0062] Shojaei, M. G. , Gutow, L. , Dannheim, J. , Rachor, E. , Schröder, A. , & Brey, T. (2016). Common trends in German Bight benthic macrofaunal communities: Assessing temporal variability and the relative importance of environmental variables. Journal of Sea Research, 107, 25–33. 10.1016/j.seares.2015.11.002

[ece35569-bib-0063] Tunberg, B. G. , & Nelson, W. G. (1998). Do climatic oscillations influence cyclical patterns of soft bottom macrobenthic communities on the Swedish west coast? Marine Ecology Progress Series, 170, 85–94. 10.3354/meps170085

[ece35569-bib-0064] Vačkář, D. , ten Brink, B. , Loh, J. , Baillie, J. E. M. , & Reyers, B. (2012). Review of multispecies indices for monitoring human impacts on biodiversity. Ecological Indicators, 17, 58–67. 10.1016/j.ecolind.2011.04.024

[ece35569-bib-0065] WFD (2000). Directive 2000/60/EC of the European Parliament and of the Council of 23 October 2000 establishing a framework for Community action in the field of water policy. Official Journal of the European Union, L327, 1–73.

[ece35569-bib-0066] WoRMS Editorial Board . (2018). World register of marine species. VLIZ. [Database]. Retrieved from http://www.marinespecies.org

[ece35569-bib-0067] Yonge, C. M. (1946). On the habits of *Turritella communis* Risso. Journal of the Marine Biological Association of the United Kingdom, 26, 377–438. 10.1017/s0025315400012182 20992402

