# Supplementary material for: Climate‐driven changes in macrobenthic communities in the Mediterranean Sea: A 10‐year study in the Bay of Banyuls‐sur‐Mer
Source: Ecol Evol. 2019 Aug 29;9(18):10483–98. doi: 10.1002/ece3.5569 (PMC6787848; doi:10.1002/ece3.5569)
Supplement: Supplementary file 1 [file ECE3-9-10483-s001.docx]

**Supporting Information for:**

Climate-driven changes in macrobenthic communities in the Mediterranean Sea: A 10-year study in the Bay of Banyuls-sur-Mer

Supplementary Table 1. Table 3 based on averaged replicate abundances: Global descriptors of sediment grain-size (D_0.5_ in µm and proportion of fine sediment) and benthic macrofauna composition (mean species richness in taxa.0.1m^-2^, mean abundance in ind.0.1m^-2^ and mean biomass in mgAFDW.0.1m^-2^).

| Sampling site | Descriptor | 2004 | 2005 | 2006 | 2007 | 2008 | 2009 | 2010 | 2011 | 2012 | 2013 |
| --- | --- | --- | --- | --- | --- | --- | --- | --- | --- | --- | --- |
| 43 | D_0.5_ | 188.2 | 196.5 | 196.0 | 198.4 | 214.4 | 210.9 | 193.0 | 223.6 | 201.6 | 202.4 |
|  | Fine sediment (%<63µm) | 2.1 | 2.2 | 0.5 | 2.2 | 0.0 | 0.0 | 0.0 | 0.0 | 0.0 | 0.8 |
|  | Species richness | 8 | 5 | 5 | 12 | 6 | 3 | 6 | 9 | 10 | 9 |
|  | Abundance | 42 | 123 | 23 | 50 | 15 | 8 | 14 | 19 | 28 | 71 |
|  | Biomass | 0.08 | 0.19 | 0.02 | 0.10 | 0.12 | 0.01 | 0.01 | 0.08 | 0.03 | 0.11 |
| 31 | D_0.5_ | 142.8 | 143.5 | 126.8 | 103.2 | 122.6 | 193.0 | 166.0 | 152.3 | 130.6 | 102.7 |
|  | Fine sediment (%<63µm) | 23.4 | 21.4 | 22.2 | 29.5 | 24.2 | 14.1 | 17.7 | 16.5 | 27.3 | 30.8 |
|  | Species richness | 11 | 11 | 6 | 10 | 7 | 9 | 8 | 9 | 13 | 21 |
|  | Abundance | 87 | 44 | 31 | 76 | 26 | 103 | 80 | 106 | 100 | 159 |
|  | Biomass | 0.09 | 0.07 | 0.03 | 0.06 | 0.04 | 0.10 | 0.08 | 0.09 | 0.11 | 0.42 |
| 26 | D_0.5_ | 68.6 | 64.5 | 64.9 | 69.3 | 72.1 | 86.6 | 74.4 | 73.3 | 64.3 | 76.7 |
|  | Fine sediment (%<63µm) | 46.2 | 49.0 | 48.6 | 45.5 | 43.7 | 34.8 | 42.2 | 43.0 | 49.1 | 40.6 |
|  | Species richness | 13 | 13 | 11 | 14 | 10 | 10 | 19 | 14 | 22 | 20 |
|  | Abundance | 102 | 89 | 73 | 79 | 34 | 47 | 102 | 132 | 200 | 167 |
|  | Biomass | 0.22 | 0.69 | 0.43 | 0.16 | 0.06 | 0.07 | 0.24 | 0.18 | 0.21 | 0.62 |
| 183 | D_0.5_ | 59.0 | NA | NA | 61.1 | 74.5 | 84.1 | 64.6 | 79.7 | 51.0 | 120.0 |
|  | Fine sediment (%<63µm) | 51.7 | NA | NA | 50.8 | 45.3 | 43.3 | 49.3 | 43.4 | 55.3 | 39.0 |
|  | Species richness | 17 | 12 | 11 | 14 | 11 | 11 | 20 | 18 | 22 | 17 |
|  | Abundance | 91 | 85 | 37 | 77 | 55 | 42 | 120 | 115 | 145 | 89 |
|  | Biomass | 0.36 | 0.54 | 0.25 | 1.11 | 0.36 | 0.58 | 0.53 | 0.80 | 0.93 | 0.45 |

D_0.5_: median grain diameter.

Supplementary Table 2. Table 4 based on averaged replicate abundances: Abundance, contribution and cumulative contributions to dissimilarities in benthic macrofauna composition for the five species most responsible for dissimilarity between the sub-clusters identified in Fig. 6b.

| Sub-clusters | Species | Average abundance | Average abundance | Contribution (%) | Cumulated (%) |
| --- | --- | --- | --- | --- | --- |
| Ia & Ib |  | Ia | Ib |  |  |
|  | *Ditrupa arietina* | 48.75 | 4.37 | 55,61 | 55.61 |
|  | *Siphonoecetes neapolitanus* | 1.3 | 1.67 | 2.85 | 58.46 |
|  | *Apseudopsis* spp. | 1.45 | 0.07 | 2.39 | 60.86 |
|  | *Urothoe grimaldii* | 1.15 | 0.27 | 2.05 | 62.91 |
|  | *Urothoe hesperia* | 1.6 | 1.37 | 1.71 | 64.62 |
| IIa & IIb |  | IIa | IIb |  |  |
|  | *Aspidosiphon muelleri* | 8.6 | 32.03 | 28.21 | 28.21 |
|  | *Ditrupa arietina* | 3.07 | 23.4 | 23.88 | 52.09 |
|  | *Owenia fusiformis* | 1.6 | 3.97 | 3.72 | 55.82 |
|  | *Anapagurus breviaculeatus* | 3.4 | 1.5 | 3.24 | 59.05 |
|  | *Turritella communis* | 0.2 | 2.37 | 2.87 | 61.92 |
| IIIa & IIIb |  | IIIa | IIIb |  |  |
|  | *Aspidosiphon muelleri* | 5.84 | 23.91 | 15.63 | 15.63 |
|  | *Turritella communis* | 2 | 12.53 | 7.73 | 23.36 |
|  | *Galathowenia oculata* | 0.84 | 10.62 | 7.18 | 30.54 |
|  | *Nephtys kersivalensis* | 5.5 | 10.11 | 5.95 | 36.49 |
|  | *Apseudopsis* spp. | 7.18 | 3.09 | 5.44 | 41.93 |
| IIIa & IIIc |  | IIIa | IIIc |  |  |
|  | *Turritella communis* | 2 | 16.1 | 13.73 | 13.73 |
|  | *Apseudopsis* spp. | 7.18 | 8.7 | 8.17 | 21.91 |
|  | *Lumbrineris latreilli* | 5.84 | 3.7 | 4.98 | 26.89 |
|  | *Aspidosiphon muelleri* | 5.84 | 2.3 | 4.48 | 31.37 |
|  | *Nephtys kersivalensis* | 5.5 | 5.4 | 3.99 | 35.36 |
| IIIb & IIIc |  | IIIb | IIIc |  |  |
|  | *Aspidosiphon muelleri* | 23.91 | 2.3 | 14.16 | 16.16 |
|  | *Turritella communis* | 12.53 | 16.1 | 7.21 | 21.37 |
|  | *Galathowenia oculata* | 10.62 | 0.6 | 6.04 | 27.41 |
|  | *Apseudopsis* spp. | 3.09 | 8.7 | 4.93 | 32.35 |
|  | *Nephtys kersivalensis* | 10.11 | 5.4 | 4.59 | 36.94 |

**Supplementary Table 3.** Table 5 based on averaged replicate abundances: Correlation coefficients of Spearman (*rho*) between climatic indices and global descriptors of benthic macrofauna (mean species richness in taxa.0.1m^-2^, mean abundance in ind.0.1m^-2^ and mean biomass in mgAFDW.0.1m^-2^). *** *p*<0.001, ** *p*<0.01, * *p*<0.05.

| Sampling site | Benthic macrofauna | NAO index | | | WeMO index | | |
| --- | --- | --- | --- | --- | --- | --- | --- |
|  |  | Annual | Spring | Winter | Annual | Spring | Winter |
| 43 | Species richness | 0.55 | 0.33 | 0.04 | 0.02 | 0.38 | 0.59 |
|  | Abundance | 0.18 | -0.33 | 0.22 | -0.01 | 0.18 | 0.66 * |
|  | Biomass | 0.31 | -0.30 | 0.38 | -0.13 | 0.09 | 0.21 |
| 31 | Species richness | 0.03 | -0.14 | 0.22 | 0.58 | 0.49 | 0.95 *** |
|  | Abundance | 0.14 | 0.19 | -0.39 | 0.38 | 0.15 | 0.59 |
|  | Biomass | -0.09 | -0.02 | -0.09 | 0.62 | 0.38 | 0.71 * |
| 26 | Species richness | -0.07 | -0.21 | -0.15 | -0.01 | 0.32 | 0.66 * |
|  | Abundance | 0.09 | -0.09 | -0.21 | 0.05 | 0.15 | 0.67 * |
|  | Biomass | -0.41 | -0.73 * | -0.32 | -0.25 | -0.26 | 0.27 |
| 183 | Species richness | 0.03 | 0.05 | -0.19 | 0.01 | 0.10 | 0.44 |
|  | Abundance | -0.04 | 0.01 | -0.16 | -0.02 | 0.01 | 0.39 |
|  | Biomass | 0.10 | 0.53 | 0.21 | 0.09 | 0.16 | 0.50 |

NAO: North Atlantic Oscillation, WeMO: Western Mediterranean Oscillation.

Supplementary Table 4. Table 6 based on averaged replicate abundances: Results from the *Best* procedure used to link benthic macrofauna abundance (mean) and biomass (mean) composition with climatic indices (computed for each integration period separately), environmental and granulometric parameters. ***: p<0.001, **: p<0.01, *: p<0.05. Black cells correspond to the parameters retained in the final model. Cells with an “X” correspond to the parameters which were not included in the model due to collinearity. Cells with “-” correspond to the parameters which were not included because missing data for 2005 and 2006.

| **Abundance** | **Annual** | | | | **Spring** | | | | **Winter** | | | |
| --- | --- | --- | --- | --- | --- | --- | --- | --- | --- | --- | --- | --- |
| **Sampling site** | **43** | **31** | **26** | **183** | **43** | **31** | **26** | **183** | **43** | **31** | **26** | **183** |
| NAO index |  |  |  |  | X | X |  | X | X | X |  | X |
| WeMO index |  |  |  |  |  |  |  |  |  |  |  |  |
| Air temperature |  |  |  |  |  |  |  |  |  |  |  |  |
| SLP |  |  |  |  |  |  | X | X |  |  | X |  |
| Wind speed |  |  |  |  | X | X |  |  |  |  |  |  |
| Precipitation |  |  |  |  |  |  |  |  |  |  |  |  |
| Rhône River water flow |  |  |  |  |  |  | X |  |  |  |  |  |
| SPM |  |  |  |  |  |  |  |  |  |  |  |  |
| Criteria 2 |  |  |  |  |  |  |  |  |  |  |  |  |
| D_0.5_ |  |  |  | - |  | X |  | - |  | X | X | - |
| Fine sediment (%<63µm) |  | X |  | - |  |  | X | - |  |  |  | - |
| **ρ** | 0.40 | 0.36 | -0.04 | 0.07 | 0.48 | 0.35 | 0.43 | 0.36 | 0.54 | 0.68 * | 0.66 ** | 0.56 * |
|  |  | | | |  | | | |  | | | |
| **Biomass** | **Annual** | | | | **Spring** | | | | **Winter** | | | |
| **Sampling site** | **43** | **31** | **26** | **183** | **43** | **31** | **26** | **183** | **43** | **31** | **26** | **183** |
| NAO index |  |  |  |  |  |  |  |  |  |  | X | X |
| WeMO index |  |  |  |  |  |  |  |  |  |  |  |  |
| Air temperature |  |  |  |  |  |  |  |  |  |  |  |  |
| SLP |  |  |  |  |  |  | X |  | X | X |  |  |
| Wind speed |  |  |  |  | X | X |  | X |  |  |  |  |
| Precipitation |  |  |  |  |  |  |  |  |  |  |  |  |
| Rhône River water flow |  |  |  |  | X | X | X | X |  |  |  |  |
| SPM |  |  |  |  |  |  |  |  |  |  |  |  |
| Criteria 2 |  |  |  |  |  |  |  |  |  |  |  |  |
| D_0.5_ |  | X | X | - |  | X | X | - |  | X | X | - |
| Fine sediment (%<63µm) |  |  |  | - |  |  |  | - |  |  |  | - |
| **ρ** | 0.52 | 0.49 | 0.23 | 0.04 | 0.27 | 0.34 | 0.60 * | 0.28 | 0.30 | 0.72 ** | 0.32 | 0.32 |

NAO: North Atlantic Oscillation, WeMO: Western Mediterranean Oscillation, SLP: Sea Level Pressure, SPM: Suspended Particulate Matter, D_0.5_: median grain diameter.

Supplementary Figure 1. Figure 5 based on averaged replicate abundances: Temporal changes in the winter WeMO index and benthic macrofauna mean species richness, mean abundance and mean biomass for each sampling site: (a, b, c) sampling site 43, (d, e, f) sampling site 31, (g, h, i) sampling site 26 and (j, k, l) sampling site 183. Symbols indicate sampling sites: 43 (circle), 31 (diamond), 26 (square) and 183 (triangle). (*) indicates significant (*p*<0.05) linear correlation.

Supplementary Figure 2. Figure 6 based on averaged replicate abundances: (a) Non-metric Multidimensional Scaling (nMDS) and (b) hierarchical cluster analysis (square-root transformed data, Bray-Curtis similarity and average group method) of macrofauna species abundance data. Symbols indicate sampling sites: 43 (circle), 31 (diamond), 26 (square) and 183 (triangle).

Supplementary Figure 3. Figure 7 based on averaged replicate abundances: Temporal changes in winter WeMO index and mean abundance of *Aspidosiphon muelleri* at sampling sites (a) 43 and (b) 183. Correlations were significant in all cases. Symbols indicate sampling sites: 43 (circle) and 183 (triangle).
